# Per- and polyfluoroalkyl substances in dog blood serum levels and semen quality

**DOI:** 10.3389/fendo.2025.1643703

**Published:** 2025-10-01

**Authors:** Jana Weiss, Josefin Engelhardt, Bodil Ström Holst, Razaw Al-Sarraj, Ida Hallberg

**Affiliations:** ^1^ Department of Environmental Science, Faculty of Science, Stockholm University, Stockholm, Sweden; ^2^ Department of Clinical Sciences, Faculty of Veterinary Medicine and Animal Science, the Centre for Reproductive Biology in Uppsala, Swedish University of Agricultural Sciences, Uppsala, Sweden; ^3^ Department of Energy and Technology, Swedish University of Agricultural Sciences, Uppsala, Sweden; ^4^ Department of Animal Biosciences, Faculty of Veterinary Medicine and Animal Science, Swedish University of Agricultural Sciences, Uppsala, Sweden

**Keywords:** PFAS, canine, reproduction, male fertility, endocrine disrupting chemicals

## Abstract

**Background:**

Growing evidence links chemical exposure to declining reproductive function in both humans and dogs. Our aim was to investigate the exposure of a wide range of per- and polyfluoroalkyl substances (PFAS) in dog serum and to investigate the association between PFAS exposure and endocrine parameters as well as semen quality.

**Method:**

Semen samples (n=65) were collected from Bernese mountain dogs during 2020. Sperm motility was evaluated under a phase-contrast microscope (100×, 200×). Total sperm count was calculated using a Bürker chamber. Sperm morphology was evaluated using standard protocols in wet preparations of semen fixed in buffered formalin and stained with carbolfuchsin-eosin. Serum was analyzed using a combined targeted and suspect screening approach for quantitative analysis of 50 PFAS. Following extraction, instrumental analysis was performed using an ultra-high-performance liquid chromatograph coupled to a Q ExactiveOrbitrap mass spectrometer. PFAS concentrations were associated with semen quality and endocrine biomarkers using Least Absolute Shrinkage and Selection Operator (LASSO) regression.

**Results:**

In all samples, PFOA, PFNA, PDFA, PFPeS, PFHxS and PFOS could be detected, although PFPeS levels were not above the quantification limit. The levels of the dominant congeners were on average (5^th^-95^th^ percentile) PFOA 0.44 (0.05-1.3) ng/g serum, PFHxS 0.39 (0.05-0.96) ng/g serum, and PFOS 2.1 (0.35-6.4) ng/g serum. Fifteen suspect PFAS congeners were identified, where perflouro-4-ethylcyclohexanesulfonate (PFECHS), H-PFOA, H-PFNA, and H-PFDA were found in > 60% of the samples. Significant associations were found between PFBS motility (β = 136.56, p = 0.03) and free androgen index (β = 0,931, p=0.02).

**Conclusion:**

For the first time, levels of a wide range of target and suspect PFAS are described in dog serum. PFAS levels in dog serum were similar to those in cats and humans, confirming that humans and pets, to a considerable extent, may share exposure to PFAS through the home environment. The study contributes to bridging the existing knowledge gap of exposure to endocrine disruptors and health effects in dogs, and thus to the research infrastructure bridging between species with the benefit of both humans and pets in a true One Health approach.

## 1 Introduction

Per- and polyfluoroalkyl substances (PFAS) are a group of synthetic compounds characterized by their strong carbon-fluorine bonds, rendering them resistant to degradation. Their pervasiveness in everyday products such as firefighting foams, non-stick cookware, and waterproof fabrics has led to their widespread distribution in the environment and subsequent global exposure (1, 2). PFAS have emerged as a class of persistent organic pollutants (POPs), listed under the Stockholm Convention (3). In recent decades, research has intensified to unravel the intricate relationship between PFAS exposure and its impact on various aspects of human health, including endocrine-disrupting properties.

Current research indicates potential links between PFAS exposure and adverse health outcomes in experimental animal models, including but not limited to immunotoxicity, hepatotoxicity, and disruption of endocrine function (4–9). Epidemiological studies in humans have highlighted associations between PFAS exposure and increased risks of certain cancers, thyroid disease, serum lipids, and impaired immune function, raising significant concerns about their long-term impact on public health (10–12). The endocrine-disruptive properties of PFAS have been attributed mainly to the activation of peroxisome proliferator-activated receptors (PPARs) (13, 14), active in glucose and lipid metabolism. However, PFAS also exert other mechanisms of toxicity. This has been illustrated in experimental animal PPAR-null models (15–17). For example, PFAS have been suggested to have an antagonistic effect on the androgen receptor (18).

Reproductive function is sensitive to hormone alterations, and emerging evidence suggests a correlation between PFAS exposure and adverse reproductive outcomes in humans, such as reduced fertility, pregnancy-induced hypertension, gestational diabetes, and low birth weight or lower IQ in offspring (10, 19–23). The mechanisms are still unclear but could be due to either direct effects of PFAS on reproductive tissue or to effects mediated by altered hormone function after endocrine-disruption by PFAS. In men, declining semen quality has been reported over the last decades (24, 25). The data on the potential association between PFAS and semen quality in men is scarce, but serum or seminal plasma concentrations of PFAS have been associated with altered reproductive hormones and semen parameters (26, 27).

Given the shared environment and common lifestyle practices between humans and companion animals, particularly dogs, there is a dual advantage of utilizing dogs not only for understanding the exposure and potential health effects in the species, but also for extrapolating findings from dogs to their owners (28). Due to widespread use, almost all humans have quantifiable levels of PFAS in their blood. Few studies have been performed on dogs, but PFAS has been detected in police- and experimental animal dogs in China (29) and in dogs near a fluorochemical industry in the US (30). PFAS have also been analyzed in dogs’ fur (31). Although less researched, similar trends as reported in humans have been observed in dogs, with deteriorating semen quality, increased incidence of testicular malformation, and reproductive cancers (32–34). Compared to humans, dogs have shorter lifespans, and especially within breeds, the variation in phenotype is low, which offers an advantage when studying complex relationships, such as between endocrine disruptors and health parameters.

In this study, we aimed to investigate the exposure of a wide range of PFAS congeners in dog blood, using both target and suspect screening. Further, we investigated the association between PFAS exposure and endocrine parameters associated with semen quality. With the advantage of using samples from one specific breed, we could minimize the variation in confounding factors such as size and conformation among the dogs.

## 2 Materials and methods

### 2.1 Dog recruitment, questionnaire and ethical considerations

The cohort has been described previously (35). In brief, during the period of March to October 2020, a cohort of privately owned Bernese Mountain Dogs (n=65) was included in the study. Owners were provided with both oral and written information detailing the study’s purpose and procedures, and subsequently granted informed consent by signing an agreement. All data handling adhered to the guidelines outlined in the General Data Protection Regulation (GDPR) and institutional guidelines. Ethical approval was obtained from the regional animal ethical committee under reference number 5.8.18-17395/2018. Preceding sample collection, owners were asked to complete a brief questionnaire (35). This questionnaire encompassed a combination of open-ended and closed questions concerning the dogs’ medical history, medication usage, and breeding records. This study uses previously collected samples (35), which is in line with the 3R principles.

### 2.2 Clinical examination and sample collection

The sampling procedure has been described previously (35). Briefly, all dogs underwent clinical examinations, including assessment of body weight (kg), palpation of the testicles, and rectal palpation of the prostate gland. Semen samples were collected via manual stimulation, whenever feasible, in the presence of a female dog in oestrus. Blood samples were drawn from the cephalic vein into tubes without additives and subsequently underwent centrifugation at 1300 × g for 10 min to retain the serum. The resulting supernatants were then stored in separate aliquots at –80°C until analysis. Sample collection took place across various clinics (n=15) spanning the northern to southern regions of Sweden. These samples were frozen and transported to the Swedish University of Agricultural Sciences for subsequent processing and analysis of biomarkers and to Stockholm University for PFAS analysis.

### 2.3 Assessment of semen quality and quantification of endocrine markers

Analysis of semen and endocrine variables has been detailed elsewhere (35). In brief, sperm motility (%) was assessed subjectively using a phase-contrast microscope at magnifications of 100× and 200×, accompanied by recording of the ejaculate volume. Subsequently, the ejaculate volume, color, sperm concentration and total sperm count was assessed at the Department of Clinical Sciences, Swedish University of Agricultural Sciences (Uppsala, Sweden). Evaluation of sperm morphology involved standard procedures, observing wet preparations fixed in buffered formalin and air-dried smears stained with carbolfuchsin-eosin. The proportion of morphologically normal spermatozoa (MNS) and abnormalities in the head, midpiece, or tail, as well as proximal droplets, were recorded.

Regarding endocrine markers, quantification had been performed in serum or seminal plasma (35). To provide a brief overview, seminal plasma alkaline phosphatase (ALP) levels were quantified in all dogs utilizing an Architect c4000 (Abbott Laboratories, Köln, Germany). The assessment of anti-Müllerian hormone (AMH) had been conducted in serum using a sandwich ELISA (AMH Gen II ELISA, Beckman Coulter, Indianapolis, IN, USA). In addition, serum analyses measured Inhibin B with a canine ELISA (AnshLabs, Wedster, TX, USA), serum hormone-binding globulin (SHBG) using a canine ELISA (MyBioSource, San Diego, CA, USA), and Insulin-like peptide 3 (INSL-3) via a quantitative sandwich ELISA (MyBioSource). Total serum testosterone concentrations were analyzed using Immulite 2000 (Siemens Healthcare Diagnostics, Erlangen, Germany). Canine prostate-specific esterase (CPSE) had been evaluated in serum using the SpeedTM CPSE immunoassay reader (Virbac, La Seyne-sur-Mer, France). Metadata, including dog characteristics and endocrine variables, are available in the **Supporting Information**, **Supplementary Table S1**.

### 2.4 Chemical analysis of PFAS in serum

The internal, recovery and native PFAS standards were all from Wellington labs, Ontario, Canada, except 11H-Perfluoroundecoic acid from Appollo Scientific, Manchester, UK (CAS:1765-48-6), 8H-Perfluorooctanoic acid (CAS: 13973-14-3) and 9H-Hexadecafluoronananoic acid (CAS: 76-21-1) from Combi-Blocks, San Diego, CA, USA, and perflouro-4-ethylcyclohexanesulfonate (PFECHS) was purchased from Wellington labs. All target analytes and corresponding internal standards used for quantifications are reported in **Supplementary Table S2**.

#### 2.4.1 Sample preparation

The sample preparation was performed according to a previous publication analyzing PFAS in human serum (36). Dog serum (0.5 g) was fortified with a mixture of internal standards (IS, 0.5 ng) in a polypropylene (PP) tube and left overnight at 4°C to equilibrate. The sample was extracted using 4 mL acetonitrile (HPLC, gradient grade ≥99.9%, Sigma Aldrich, Darmstadt, Germany), vortexed, and ultrasonicated for 15 min. After centrifugation (1500 × g, 5 min), the supernatant was transferred to a new PP tube and the extraction repeated once. The extract was concentrated to 1 mL under a gentle stream of nitrogen gas and transferred to a pre-weight Eppendorf tube containing 25 mg ENVI-Carb (bulk packing Superclean™, Supelco, Darmstadt, Germany) and 50 µL glacial acetic acid (p.a. ACS reagent ≥99.8%, Sigma Aldrich). The weight of the extract was noted. The tube was vortexed and centrifuged (7300 × g, 10 min) and 500 µL of the supernatant was transferred to another Eppendorf tube, in which the recovery standard (RS, 0.5 ng) and 4 mmol/L ammonium acetate (EMSURE^R^ ACS, Reag. Ph Eur, Merck KGaA, Darmstadt, Germany) in MilliQ water (200 µL) were added. The samples were stored at -20°C until instrumental analysis.

#### 2.4.2 Instrumental analysis

The instrumental analysis was performed according to a previously published method (36). The samples were analyzed using a Dionex UltiMate™ 3000 Ultrahigh performance liquid chromatograph (UPLC, Thermo Fisher Scientific Inc.) combined with a Q Exactive™ HF hybrid Quadrupole-Orbitrap™ mass spectrometer (MS) (Thermo Fisher Scientific Inc.). A BEH C18 column (2.1 x 50 mm, 1.7 µm particle size, Waters) combined with a guard column BEH C18 (2.1 x 5 mm, 1.7 µm particle size, Waters) were used for chromatographic separation. To avoid background contamination from the mobile phases, an isolator column XBridge™ C18 (2.1 x 50 mm, 3.5 µm particle size, Waters) was mounted before the injector. The buffer of the mobile phases was 2 mmol/L ammonium acetate in either 95% pure MilliQ water and 5% acetonitrile (mobile phase A) or 95% acetonitrile and 5% MilliQ water (mobile phase B). An injection volume of 5 µL and a flow rate of 0.4 mL/min were used. The initial ratio of the mobile phases was 90% A and 10% B. After 0.5 min, mobile phase B was increased linearly up to 99% during 7.5 min. The ratio of 100% was held for 3 min and then switched to 90% A for column equilibration for two min. The total time of each run was 13 min.

The MS was run in negative mode, in full scan (150–2000 Da), using data-dependent acquisition. A list of 87 target analytes was used (including 22 internal standards and 2 recovery standards, **Supplementary Table S2**) together with an inclusion list of 311 analytes for suspect screening (37). M8PFOS was used as the recovery standard for the perfluoralkyl sulfonic acids (PFSAs) and M8PFOA was used as the recovery standard for the PFCAs. The target and suspect data processing were done using Tracefinder version 4.1 (Thermo Fisher Scientific Inc.) and Xcalibur Qualbrowser for MS^2^ spectra (Thermo Fisher Scientific Inc.).

#### 2.4.3 Quality assurance/quality control for PFAS analysis in serum

The sample preparation was conducted in four batches, each comprising 17 individual samples. For each batch, two to three method blank samples (empty tubes) and one in-house reference sample of human blood were included for quality control. In addition, two sampling blanks were created by using the serum collection tubes and syringe. An 8-point calibration curve was created with a concentration span of 0.02–15 ng/mL, using linear curve fit with 1/x weighting. The calibration curve was injected before and after the samples in the instrumental analysis.

An analyte was considered below the limit of detection (LOD) if there was no well-shaped bell curve with a minimum peak/noise ratio of more than 3. The limit of quantification (LOQ) was set to the lowest calibration point where a well-shaped peak was seen. If an analyte was detected in the blank samples, the method LOQ was calculated as the mean concentration in blanks plus three times the standard deviation. Generally, the background contamination was lower than the LOQ based on the lowest calibration curve. Only two analytes, PFHxA and PFOS were found in blank samples, in 33% and 56% of the nine blank samples, respectively. This increased the method LOQ to be 0.37 ng/g for PFHxA and 0.16 ng/g for PFOS. The method LOQs for all validated analytes are reported in **Supplementary Table S3**.

During suspect screening, if a suspect feature was found in any blank sample, the feature was disregarded and not processed further. Features detected in the solvent peak (<1 min) were excluded. Additionally, the threshold for the isotopic pattern score (IPS) was set to above 60% and the mass-to-charge ratio <5 ppm.

A recovery test was performed prior to the batch analysis of the samples. Two triplicates of serum reference samples fortified with native standards (0.5 ng and 1 ng, respectively) were analyzed. Of the 65 PFAS target analytes evaluated in the project, 9 compounds, along with all branched isomers (n=11) were not available in the native standard mixture for evaluation. Consequently, 45 PFAS were evaluated for the analytical method. Of these, 35 PFAS showed satisfactory recovery in the method (50-150%) (**Supplementary Table S3**). Those PFAS that could not be validated in the recovery test were semi-quantified using a structurally similar compound in the calibration curve. In summary, 55 PFAS were included in the target analysis of the dog serum.

Only compounds found above the LOD (detection frequency [DF] > 0%) in at least one dog serum sample are reported in the result tables (**Supplementary Table S4**). These are 24 validated PFAS and 7 semi-quantified PFAS (PFPeS, PFHxS-br, PFHpS, PFHpS-br, PFOS-br, PFNS, PFDS-br). Further, 15 PFAS were quantified (quantification frequency [QF] > 0%) in dog serum (**Supplementary Table S4**).

For quality assurance, three samples of certified reference material (CRM) for human blood analysis (NIST 1957) were analyzed and compared to reported values in literature and from NIST to demonstrate accuracy. The results were in good agreement with other reported values (**Supplementary Table S5**).

### 2.5 Data processing and statistical methods

For the statistical evaluations, the summation of the branched and linear congeners of the target PFAS was used. Values below LOD were set to zero, while values above LOD but below LOQ were replaced by LOQ/2. Free testosterone was estimated using the free androgen index (FAI), calculated as the ratio between testosterone and SHBG. The percentage morphologically normal spermatozoa (MNS) was calculated by subtracting the sum of percentage of spermatozoa without defects (counted by wet preparation with formol saline solution) and percentage of spermatozoa with distal cytoplasmic droplets (wet preparation with formol saline) with the percentage of spermatozoa with pathological heads (smear stained with William’s stain). A percentage range of morphologically normal spermatozoa was then obtained. The percentage of MNS was calculated by adding the higher and the lower range and then divided by two. Total sperm count was calculated by multiplying the volume of the ejaculate with the concentration and presented as × 10^6^.

The relationships between PFAS exposure and semen quality, and between PFAS exposure and endocrine biomarkers, were investigated. PFAS that were above LOQ in more than 25% of the samples (PFOA, PFNA, PFDA, PFBS, PFHxS, and PFOS) were included, as well as the summation of PFAS (total PFAS). Age and weight were included as potential confounders, identified through a directed acyclic graph (DAG; **Supplementary Figure S1**). Weight was added as a proxy for body-mass-index, BMI, or body condition score (BCS), as the variation in size was limited because the cohort consisted of dogs from one specific breed. Previous research has demonstrated associations between endocrine markers in serum and semen parameters (35). Consequently, for sperm-related outcomes, endocrine markers were considered potential mediators and investigated as outcomes, while age and weight were identified as potential confounders.

First, the correlations between individual target PFAS detected in >25% of the samples, total PFAS, endocrine biomarkers, and semen quality were investigated using Spearman’s correlation. A p-value <0.05 and correlation coefficient (ρ) > ± 0.25 were considered indicative of a significant association.

To further examine the associations between sperm quality parameters (motility, total sperm count, and MNS), hormonal biomarkers (AMH, Inhibin, FAI, INSL3, ALP, and CPSE), and exposure to PFAS, multivariate regression analyses were conducted using both multiple linear regression (MLR) and Least Absolute Shrinkage and Selection Operator (LASSO) regression (38). Age and weight were included as potential confounders. MLR was employed to estimate the effects of explanatory variables on each response variable. Where assumptions of normality and homoscedasticity were not met, response variables were log- or square root–transformed, and Box-Cox transformations were applied where appropriate. Model diagnostics, including residual analysis and the detection of influential observations, were used to validate model assumptions. LASSO regression, a penalized regression technique that shrinks less relevant coefficients toward zero, was used to enhance model selection and reduce the risk of overfitting, particularly in the context of potentially correlated predictors (38). Initially, both MLR and LASSO models included age, weight, and total PFAS concentration (total PFAS) as predictors. In a subsequent analysis, the predictor set was expanded to include individual PFAS congeners (PFBS, PFDA, PFHxS, PFNA, PFOA, and PFOS). All models were applied to both sperm quality and hormone outcome variables. Statistical significance was defined as p < 0.05, and all analyses were performed using Minitab (Minitab version 19.2020.1 (64 bit)) and R version 4.4.1 (R Core Team, 2025), LASSO regression was performed using the glmnet package (version 4.1.8).

In addition, for the discussion, PFAS exposure in cats, humans, and dogs from Sweden were compared using raw data on cat serum sampled in 2013-2014 (39), and human samples from 2020 (40), both from Stockholm region, Sweden. The concentrations of PFAS analyzed in all three species (PFOS, PFOA, PFNA, PFHxS, PFHpA, PFDoDA, and PFDA) were compared using one-way ANOVA (car package, R 4.3.1). A p-value <0.05 was considered significant.

## 3 Results

### 3.1 PFAS exposure assessment

#### 3.1.1 Target analysis

Statistics on PFAS levels in dog serum are reported in **Table 1** and details on individual samples are given in **Supplementary Table S4**. In all dog serum samples, PFOA, PFNA, PFDA, PFPeS, PFHxS, and PFOS could be detected (detection frequency, DF = 100%), although PFPeS levels were all below LOQ. The most frequently quantified PFAS analytes were PFOA, PFNA, PFBS, PFHxS (linear), and PFOS (linear and branched) with a quantification frequency (QF) >70%. The highest average levels (5-95^th^ percentile) of PFAS were 0.44 ng PFOA/g serum (0.05-1.3), 0.39 ng PFHxS/g serum (0.05-0.95), and 2.1 ng PFOS-tot/g serum (0.35-6.4). Maximum levels reported in individual serum samples were 17 ng 8:2 FTSA/g, 12 ng PFOS-tot/g, 5.3 ng PFOA, and 4.5 ng PFHxA/g serum (**Table 1**).

**Table 1:** Average, median, 5th and 95th serum levels (ng/g serum) of PFAS in dogs (n=65), together with the detection frequency (DF) and quantification frequency (QF).

Target analyte	Average	Median	5th	95th	Max	DF (>LOD)	QF (>LOQ)
PFBA	0.02	0.02	<LOQ	<LOQ	0.11	36%	2%
PFPeA	0.01	0.014	<LOQ	<LOQ	0.089	55%	5%
PFHxA	0.26	0.18	<LOQ	0.63	4.5	74%	11%
PFHpA	0.037	0.051	<LOQ	<LOQ	0.18	70%	2%
PFOA	0.44	0.22	0.051	1.3	5.3	100%	79%
PFNA	0.24	0.15	0.032	0.74	1.1	100%	95%
PFDA	0.088	0.051	0.051	0.23	0.35	100%	29%
PFUnDA	0.071	0.051	0.051	0.21	0.30	98%	15%
PFDoDA	0.049	0.051	0.051	<LOQ	<LOQ	97%	0%
PFTriDA	0.038	0.051	<LOQ	<LOQ	<LOQ	76%	0%
PFTeDA	0.0048	<LOQ	<LOQ	<LOQ	<LOQ	33%	0%
PFHxDA	0.029	0.051	<LOQ	<LOQ	<LOQ	58%	0%
PFBS	0.023	0.015	0.0041	0.072	0.091	95%	74%
PFPeS	0.05	0.051	0.051	<LOQ	<LOQ	100%	0%
PFHxS	0.39	0.33	0.051	0.95	1.7	100%	82%
PFHxS-br	0.0061	<LOQ	<LOQ	<LOQ	<LOQ	12%	0%
PFHpS	0.048	0.051	0.013	<LOQ	<LOQ	94%	0%
PFHpS-br	0.038	0.051	<LOQ	<LOQ	<LOQ	76%	0%
PFOS	1.5	0.93	0.27	4.1	9.9	100%	98%
PFOS-br	0.54	0.4	0.051	1.4	2.3	98%	91%
PFOS-tot	2.1	1.4	0.35	6.4	12	98%	98%
PFNS	0.0031	<LOQ	<LOQ	<LOQ	<LOQ	6%	0%
PFDS	0.00066	<LOQ	<LOQ	<LOQ	<LOQ	5%	0%
PFDS-br	0.00022	<LOQ	<LOQ	<LOQ	<LOQ	2%	0%
4:2 FTSA	0.079	0.08	<LOQ	0.17	4.1	39%	18%
8:2 FTSA	0.29	0.29	<LOQ	0.14	17	29%	6%
9Cl-PF3ONS	0.0014	0.0014	<LOQ	<LOQ	<LOQ	33%	0%
EtFOSA	0.00019	0.00019	<LOQ	<LOQ	<LOQ	5%	0%
EtFOSAA	0.0012	0.0012	<LOQ	0.0031	0.05	6%	3%
FOSAA	0.00069	0.0007	<LOQ	0.0065	<LOQ	11%	0%
MeFOSAA	0.00063	0.00057	<LOQ	0.0041	<LOQ	15%	0%
NADONA	0.0005	0.00051	<LOQ	0.0041	<LOQ	12%	0%

#### 3.1.2 Suspect screening

Of 311 PFAS in the inclusion list for suspect screening, 15 could be tentatively identified in at least one sample (**Table 2**). The confidence level for PFAS identification was between 4 (unequivocal molecular formula) and 2a (probable by library spectrum match) according the definition presented by Charbonnet and co-workers (41). Perflouro-4-ethylcyclohexanesulfonate (PFECHS) was detected in 97% of the samples, followed by the hydrogen-substituted H-PFOA, H-PFNA. H-PFDA was found in more than 60% of the samples.

**Table 2:** Detected suspect features (abbreviation and chemical name), with detection frequencies (DF), chemical formula (neutral), theoretical mass to charge (m/z), m/z difference within the samples (delta m/z), retention time (RT), isotopic pattern score (IPS) and level of confirmation according to Charbonnet et al., 2022 (41).

Abbreviation	From inclusion list	Chemical formula	Theoretical m/z	Delta m/z [ppm]	Results this study			Confirmation	Detected in humans			Confirmation
	Chemical name				DF	RT	IPS (range)		DF	RT	IPC (mean)	
10:2 FTSA	10:2 Fluorotelomer sulfonic acid	C12F21SO3H5	626,95513	<1,1	4,5%	5,6 ± 0,04	100%	Level 4				
12:3 FTCA	12:3 Fluorotelomer carboxylic acid	C15F25O2H5	690,9818	<4,3	3,0%	5,6 ± 0,1	70-84%	Level 4				
H-PFOA	H-substituted PFCA n=8	C8F14H2O2	394,97475	<2,5	74%	3,5 ± 0,4	68-100%	Level 2a	28%	3,4 ± 0,2	100%	Level 1b
H-PFNA	H-substituted PFCA n=9	C9F16H2O2	444,97207	<3,4	70%	4,2 ± 0,1	62-100%	Level 2a	12%	3,9 ± 0,2	80%	Level 1b
H-PFDA	H-substituted PFCA n=10	C10F18H2O2	494,96899	<3	61%	4,5 ± 0,1	100%	Level 2c	62%	3,9 ± 0,2	100%	Level 2c
H-PFDoDA	H-substituted PFCA n=12	C12F22H2O2	530,9664	<2,8	21%	5,4 ± 0,3	100%	Level 2c	12%	4,8 ± 0,2	100%	Level 2c
mOPFLSA n=3	Multiple ether-substituted perfluoroalkyl (linear) sulfonic acids, n=3	C7HF15O5S	480,92324	<1,9	6,0%	4,9 ± 0,1	75-100%	Level 4	17%	4,6 ± 0,1	98%	Level 4
ClOPFLSA n=6	Cl-substituted,ether-substituted perfluoroalkyl (linear)sulfonic acids, n=6	C8HF16O4SCl	530,89558	<0,5	1,5%	5,4 ± 0,1	76-100%	Level 4				
ClPFLCAs n=7	Cl-substituted perfluoroalkyl (linear) carboxylic acids, n=7	C8HF14O2Cl	428,93688	<1,5	27%	4,4 ± 0,1	21 >60%	Level 3d	27%	4,1 ± 0,2	83%	Level 3d
ClPFLCAs n=8	Cl-substituted perfluoroalkyl (linear) carboxylic acids, n=8	C9HF16O2Cl	478,93368	<1,8	9,0%	4,7 ± 0,1	66-97%	Level 3d	23%	4,4 ± 0,1	81%	Level 3d
ClPFLCAs n=9	Cl-substituted perfluoroalkyl (linear) carboxylic acids, n=9	C10HF18O2Cl	528,93049	<1	12%	5,1 ± 0,1	66-100%	Level 3d	35%	4,8 ± 0,1	85%	Level 3d
PFECHS (d/C PFSA n=8)	Double bond/cyclic PFSAs n=8	C8F15SO3H	460,93341	<2	97%	4,5 ± 0,4	63-100%	Level 2a	100%	4,3 ± 0,2	100%	Level 1b
dPFAmCACEs n=7	Double bond perfluoroalkyl amine carboxylic acids/carboxyl esters, n=7	C15F28O2N	757,94874	<2,5	8,0%	6,7 ± 0,01	91-100%	Level 4				
eecec PFSA n=8	Enol ether-/cyclic ether-/carbonyl-perfluoroalkyl sulfonates n=8	C8F15SO4H	476,92833	<2,6	29%	4,6 ± 0,3	68-100%	Level 4	95%	4,5 ± 0,2	93%	Level 4
PFAmCEs_i n=4	Perfluoroalkyl amine carboxyl esters_i, n=4	C14H2F26O5N	757,95233	<4,2	6,0%	6,7 ± 0,01	75-97%	Level 4				

The results are compared to reported suspects in human blood samples from 2020, Engelhardt et al., 2025 (42).

#### 3.1.3 Correlation between PFAS target and suspect compounds

In general, a significant correlation was found between individual PFAS compounds (**Supplementary Figure S2**). The relatively newly identified suspect H-PFCA in human serum [**Table 2**, H-PFOA, H-PFNA, H-PFDA, and H-PFDoDA (42)] correlated with each other. Suspect PFECHS correlated with all target PFAS except PFOA, but not the other suspects (42).

### 3.2 Association between semen quality, endocrine biomarkers and PFAS levels in blood

Spearman’s correlation indicated a negative correlation between semen quality and age (**Supplementary Figure S2**). There were also some correlations observed between individual PFAS congeners and endocrine biomarkers and/or semen quality (**Supplementary Figure S2**). Only PFBS significantly correlated with higher age (p<0.05). The correlation between PFAS and semen quality was in general positive (positive correlation with MNS while negative correlations with proportion specific defects, **Supplementary Figure S2**).

**Figure 1:**
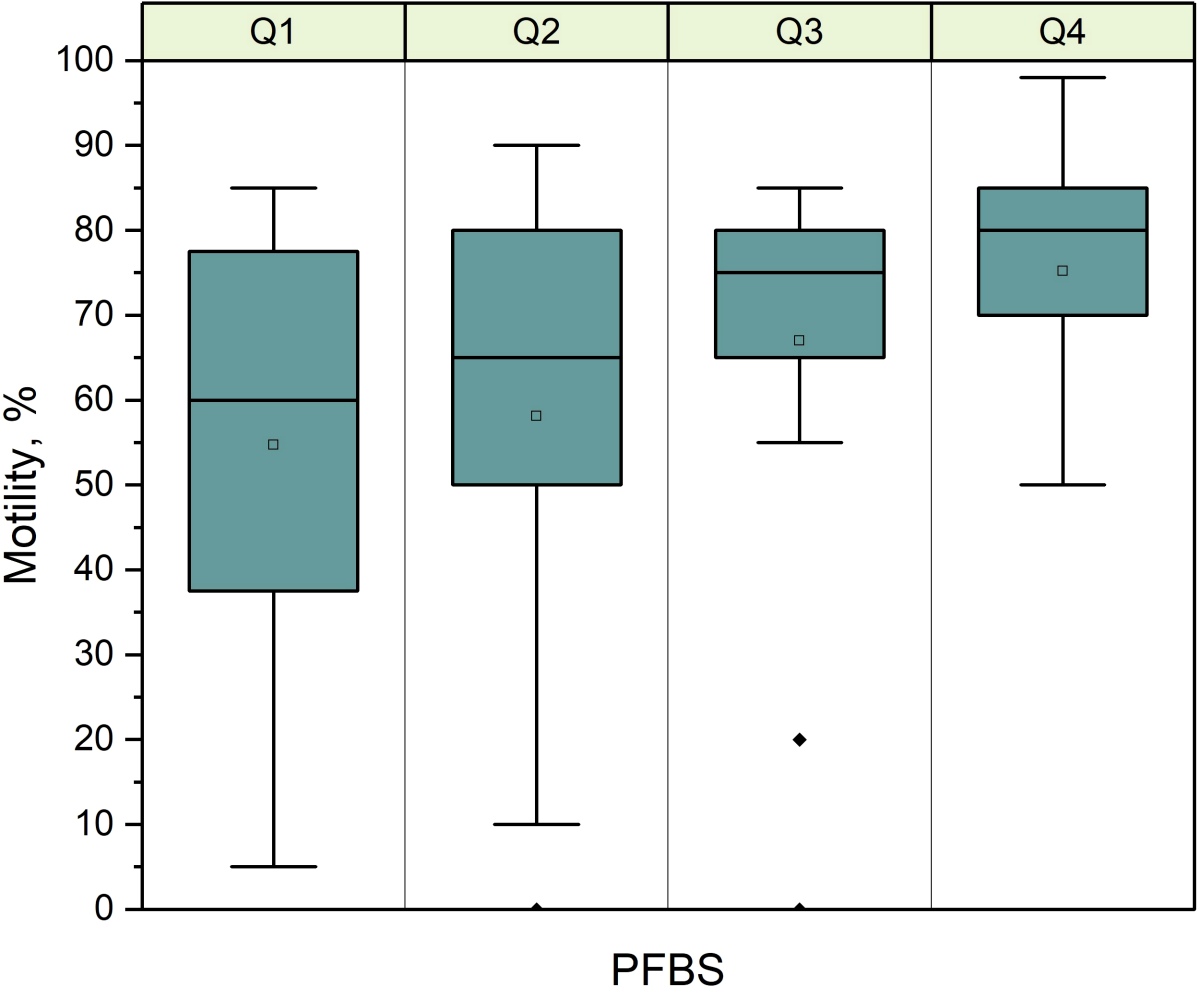
Samples divided by quartiles of PFBS exposure vs sperm motility (%). There was a significantly increased motility (%) with higher PFBS exposure (p=0.03).

The association between PFAS, endocrine biomarkers and semen quality was investigated considering the possible confounders age and weight. Multiple linear regression models (MRLs, **Supplementary Table S7**) and LASSO regression (**Tables 3**, **4**) were used to evaluate these relationships. Age emerged as a frequent and important predictor for semen quality (**Tables 3**, **4**). PFBS (β = 136.56, p = 0.026) was identified as a relevant predictor for motility (**Figure 1**). For the proportion of MNS, PFBS (β = 24.88, p = 0.086) was included in the model, although only age was statistically significant. Total PFAS exposure was not a significant predictor, and no variables were selected as predictive for total sperm count.

**Table 3:** LASSO regression analysis investigating the association between PFAS levels in serum, semen quality and possible confounders Age, Weight. Estimates (p-values), where NA indicate variables with no significant predictive value.

Response	Intercept	Age	Weight	PFBS	PFDA	PFHxS	PFNA	PFOA	PFOS
Motility	74,83	-0,27 (0.005)	NA	136,56 (0.026)	NA	NA	NA	NA	NA
Total sperm count	1,15	NA	NA	NA	NA	NA	NA	NA	NA
MNS	55,454	-0,26 (0.018)	NA	24,88 (0.086)	NA	NA	NA	NA	NA
AMH	5,55	NA	NA	NA	NA	NA	NA	NA	NA
Inhibin	0,18	-0,01 (0.89)	NA	210,16 (0.055)	NA	NA	NA	NA	NA
FAI	0,061	-0,0008 (0.01)	NA	0,931 (0,015)	NA	NA	NA	NA	NA
INSL3	144,49	-0,63 (0.54)	NA	-1269,5 (0.36)	NA	NA	NA	NA	NA
ALP	146217,2	-1599 (0.53)	NA	6685544 (0.051)	NA	NA	NA	NA	NA
CPSE	182,71	3,48 (<0.0001)	NA	74,42 (0.09)	NA	NA	NA	NA	NA

PFDA was selected as predictive for FAI, PFNA for INSL-3 and PFOS for CPSE but excluded when OLS was performed.

**Table 4:** LASSO regression analysis investigating the association between total PFAS exposure semen quality, endocrine biomarkers including possible confounders Age, Weight. Estimates (p-values), where NA indicate variables with no significant predictive value.

Response	Intercept	Age	Weight	PFAS_tot
Motility	77.08	-0.301(0.0016)	NA	NA
Total sperm count	1.1522	NA	NA	NA
MNS	55.844	-0.267(0.01)	NA	NA
AMH	5.55	NA	NA	NA
Inhibin	33.11	NA	0.976 (0.0499)	NA
FAI	0.153	0.00098(0.0397)	NA	NA
INSL3	141.1	NA	NA	NA
ALP	208564.7	NA	NA	NA
CPSE	328.34	3.467(0.000002)	-4.33(0.29)	7.17 (0.066)

Several significant associations were observed between age and endocrine biomarkers, indicating that age is an important predictive variable (**Tables 3**, **4**). LASSO regression did not select weight as an important predictor for endocrine biomarkers.

In the investigation of association between PFAS and endocrine biomarkers, PFBS was selected as a significant positive predictor for FAI (β = 0,931, p=0,015). Additionally, PFBS was selected as predictor for ALP concentrations in ejaculates (β = 6685544, p = 0.051), Inhibin B (β = 210,16, p=0.055), INSL-3 (β = -1269,5 p= 0.36) and CPSE (β =-1269,5, p=0,36) although none of these associations reached statistical significance. PFOS was selected by LASSO as a predictor for CPSE but not retained after ordinary least square (OLS) adaptation (β = 74.42, p = 0.09). Similarly, total PFAS burden was included in the model for CPSE by LASSO regression, although it did not reach statistical significance (β= 7.725, p=0.066). There were no significant predictors for AMH.

Multiple linear regression showed no significant association between PFAS exposure and semen quality, but a significant positive association was observed between total PFAS exposure and CPSE (p=0.03, **Supplementary Table S7**).

## 4 Discussion

**Figure 2:**
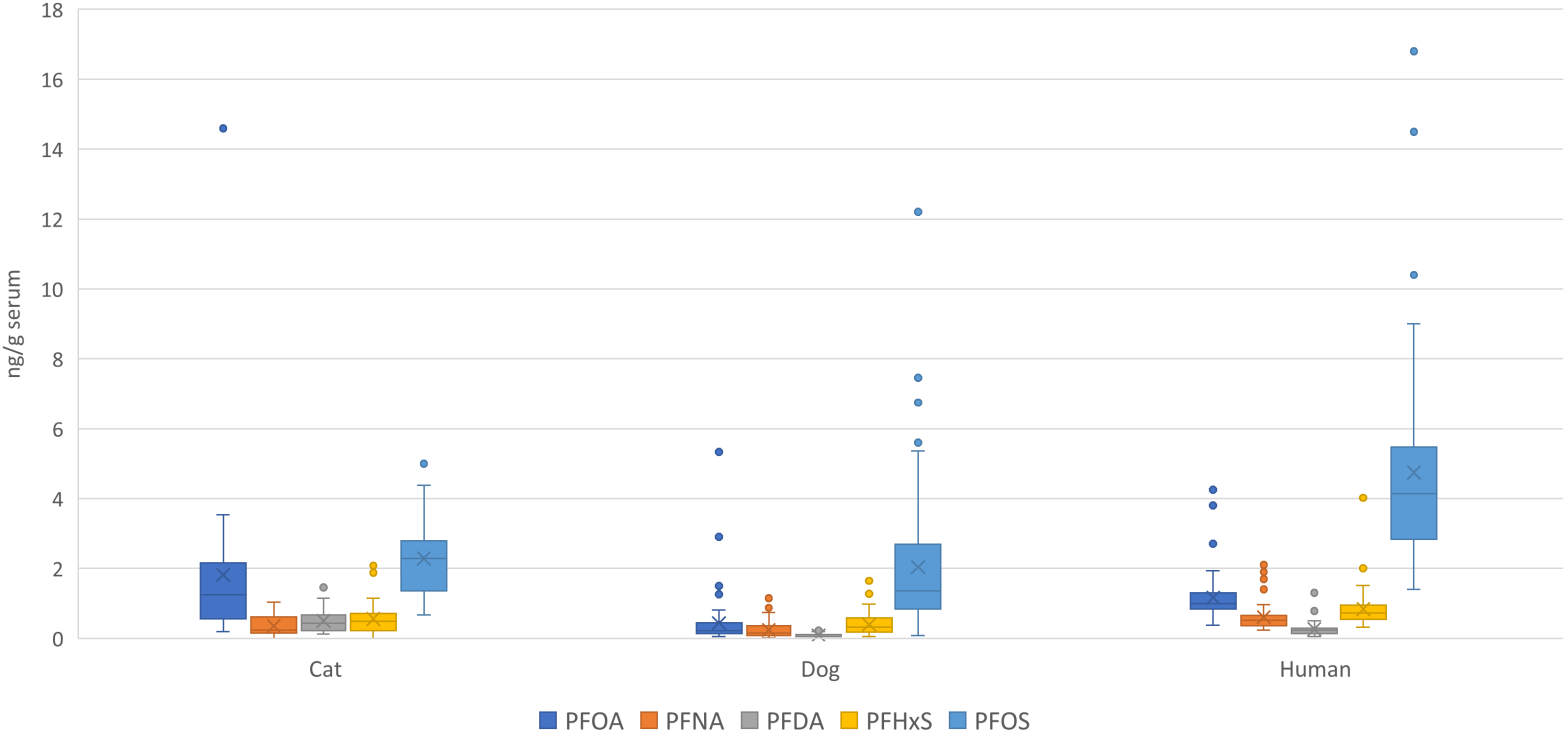
PFAS levels (average and min and max error bars) in blood serum determined in dogs (this study), cat (39) and humans (40) from Sweden.

In this study we present data on a wide range of target and suspect PFAS in sera from a population of Bernese Mountain dogs in Sweden sampled during 2020. By comparing PFAS levels between dogs (this study), cats (39) and humans (40, 42) from Sweden, we could show that the species have a similar exposure, coming from the shared indoor environment (**Figure 2**). The profiles in all species were dominated by PFOS, followed by PFOA and PFHxS. Generally, cats were more exposed to the PFCAs than dogs and humans, and humans had a higher concentration of PFOS than cats and dogs. The higher concentrations of PFOS in humans compared to both cats and dogs are likely due to the fact that PFOS correlates with age in humans (40). Except for PFBS, this study did not observe a correlation between PFAS and age in our population, which could be explained by the shorter lifespan of dogs compared to humans. Similarly, no associations have been observed between exposure and age in cats (39). Differences in exposure between species can be attributed to variations in diet. In humans, diet is an important source of PFAS exposure (43), and specific food products have been associated with exposure to certain congeners (44–46). In dogs, dietary exposure appears to account for a smaller proportion of the total exposure (29).

In humans, several adverse health outcomes have been associated with PFAS exposure, resulting in revisions of the legislation to counteract risks. There is a gap in knowledge about possible adverse health outcomes associated with exposure to pets (47), which calls for more research to understand the contribution of PFAS in the development of disease in pets.

Infertility is a concern within the canine population with both genetic and environmental etiology. A negative trend in semen quality in dogs has been suggested (32), similar to the one reported in humans (24). Parallel trends in both species point toward potential environmental influences, such as exposure to endocrine-disrupting chemicals from our shared environment. However, our results do not suggest that PFAS exposure is a major contributor to the decreased semen quality observed in dogs. In humans, conflicting results have been reported regarding associations between PFAS exposure and semen parameters or reproductive hormone levels (26, 27), possibly reflecting the complexity of infertility. In dogs, data remain limited. By focusing on a single breed in the current study, we minimized the confounding from factors such as genetic variability, size, and conformation, which are known to affect semen quality (48, 49). Nonetheless, unmeasured confounders, including potential breed-related predispositions, cannot be excluded. More research is needed across diverse populations to clarify the possible contribution of PFAS exposure to reproductive outcomes, particularly concerning endocrine function.

We observed a potential positive association between PFBS exposure and male reproductive parameters, specifically increased sperm motility and elevated FAI. These findings should be interpreted with caution, given the limited and inconsistent literature. For example, a study in 740 healthy men reported a negative association between PFBS and sperm count, but no significant association with motility or total PFAS burden (50). Another study found a negative correlation between PFBS and motility, with stronger associations seen for PFAS concentrations in semen compared to serum (51). Such findings suggest that individual PFAS congeners may exert effects independent of total exposure, in line with the results from the current study.

The potential mechanisms by which PFAS may influence male reproductive function remain unclear. Conflicting results have been reported, with PFAS mixtures inversely associated with estradiol and E2/testosterone ratios in young men (50). In contrast, positive associations between PFOA/PFOS and testosterone levels have been found in highly exposed populations (52). Experimental studies in other species also report inconsistent effects on testosterone regulation (53). PFAS effects on biochemical parameters have also been noted in canine studies (29, 30), although we did not assess biochemical parameters in the present study, which may limit our mechanistic interpretation. Taken together, these conflicting findings suggest that PFAS may affect endocrine pathways with potential implications for semen quality, though a clear understanding is still lacking.

The discrepancy between MLR and LASSO modelling in our analysis further reflects this complexity. LASSO identified PFBS and PFOS as potential predictors, whereas these associations were not consistently significant in traditional MLR models. This difference is likely attributed to issues such as multicollinearity among predictors or small effect sizes, both of which can obscure true associations in standard regression analyses.

Finally, PFAS-related effects may extend beyond reproductive hormones. Our study found a positive association between total PFAS exposure and CPSE. There is a positive association between CPSE and prostatic size in dogs (54), where a large prostate indicates prostate hyperplasia (55). The development of prostatic hyperplasia in dogs and humans are similar, which means that comparative aspects are of interest. In humans, associations have been reported between PFOS and elevated PSA levels, as well as PFAS mixtures and prostate hyperplasia (56). Although prostatic hyperplasia is not equivalent to prostate cancer risk in dogs, findings from human occupational cohorts suggest that PFAS exposure may influence DNA methylation at prostate cancer risk loci (57), warranting further investigation.

Overall, our findings contribute to a growing and complex field of research. Inconsistencies between studies, species, and analytical approaches, combined with uncertainties in biological mechanisms, underscore the need for further integrated research to better understand the effects of PFAS on male reproductive health. This study strengthens the foundation for a cross-species research infrastructure, supporting the One Health perspective. Furthermore, the research on dog cohorts on one specific breed, or larger cohorts on mixed breeds, could be valuable for investigating other health outcomes in males or females related to exposure to endocrine disruptors.

## 5 Conclusions

This study presents the first data on a wide range of target and suspect PFAS congeners in dog blood in Sweden, and associates the levels with semen quality and endocrine biomarkers and a biomarker for prostatic size. Our finding supports the notion that humans, dogs, and cats share exposure to PFAS through the home environment, highlighting the advantage of studying associations between exposure and adverse health effects in these species. The study is a significant contributor to the existing knowledge gap on exposure to endocrine disruptors and health effects in dogs, contributing to the research infrastructure bridging between species (58) with the benefit of both humans and pets in a true One Health approach.

## Data Availability

The original contributions presented in the study are included in the article/**Supplementary Material**. Further inquiries can be directed to the corresponding author.
